# Children’s Health: Flu, Fetuses, and Schizophrenia

**Published:** 2004-12

**Authors:** Julie Wakefield

Pregnant women who contract the flu may increase the risk that their child will develop schizophrenia later in life, according to a recent addition to a growing body of research along these lines. The study, published in the August 2004 *Archives of General Psychiatry*, “is not definitive but is the strongest evidence thus far that a prenatal virus may be a risk factor [for schizophrenia],” says lead investigator Ezra Susser, head of epidemiology at Columbia University’s Mailman School of Public Health.

“Influenza infection during pregnancy appears to be a risk factor,” agrees Johns Hopkins University neurovirologist Robert Yolken, who adds it is probably one of many risk factors for developing schizophrenia. The severe mental illness, which usually involves delusions, hallucinations, and disordered thinking, affects about 1% of the U.S. population. The Mailman team’s work is part of a larger study designed to examine prenatal infection and such factors as father’s age and prenatal exposure to chemicals in influencing schizophrenia in adulthood.

The Mailman team looked for influenza antibody in archived blood samples from 64 women whose children developed schizophrenia as adults and a control group of 125 women whose children did not develop the disorder. The samples were collected as part of the Child Health and Development Study, which collected blood samples from more than 12,000 mothers of children born between 1959 and 1967 and followed the children’s development into adulthood.

The risk of schizophrenia was tripled when the mother had the flu during the first half of pregnancy and increased sevenfold if exposure occurred in the first trimester. The overall risk is small, however. The findings suggest that about 97% of children born to women who got the flu while pregnant will not develop schizophrenia.

Although researchers do not know the mechanism of action, the Mailman team speculates that antibodies released by the mother’s immune system may affect the developing brain. But direct effects from the flu virus are also possible.

Researchers believe schizophrenia may result from a combination of genetic and environmental factors, including complications during delivery and exposure to the herpes simplex virus type 2 and to rubella virus during pregnancy. “It may not be just one virus,” Yolken says. “And [the key environmental factor] may vary from population to population, as genetic factors likely play a role.” Moreover, different strains of herpes or flu viruses may play greater or lesser roles.

Until more study is completed, the Mailman team still advocates that pregnant women get the flu shot. Susser says, “The very safest thing would be to get vaccinated against the flu virus before becoming pregnant.”

## Figures and Tables

**Figure:**
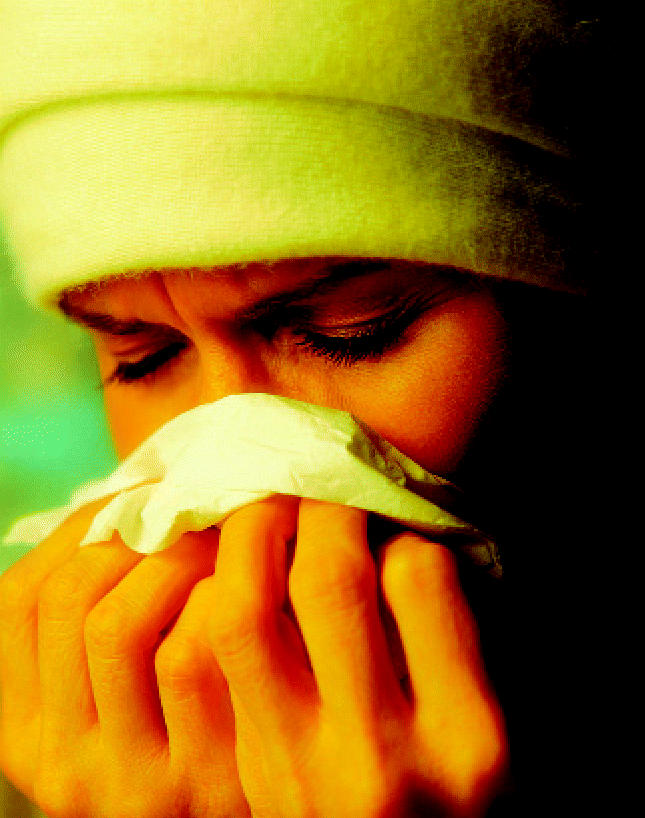
**A cold now, catastrophe later.** Having the flu while pregnant may pave the way for future schizophrenia in children.

